# Diffuse eruptive squamous atypia, also known as eruptive keratoacanthoma: Unique case presentation

**DOI:** 10.1016/j.jdcr.2024.04.043

**Published:** 2024-05-10

**Authors:** Julia Griffin, Mackenzie Asel

**Affiliations:** a Creighton University School of Medicine, Omaha, Nebraska; b Department of Dermatology, University of Wisconsin, Madison, Wisconsin

**Keywords:** acitretin, eruptive keratoacanthoma, eruptive squamous atypia, intralesional fluorouracil, squamous cell carcinoma mimic

## Introduction

Cutaneous squamous cell carcinoma (SCC) is the second most common type of malignancy and accounts for about 20% of nonmelanoma skin cancers.^1^ A rare mimicker of cutaneous SCC is eruptive squamous atypia (ESA) which is often incorrectly diagnosed and treated as SCC. This unique disease presents idiopathically or due to koebnerization as atypical hyperkeratotic squamous proliferations commonly on the lower extremities. It is important to review this particular diagnosis so patients are accurately diagnosed and treated.^2^, ^3^, ^4^

## Case report

**Fig 1:**
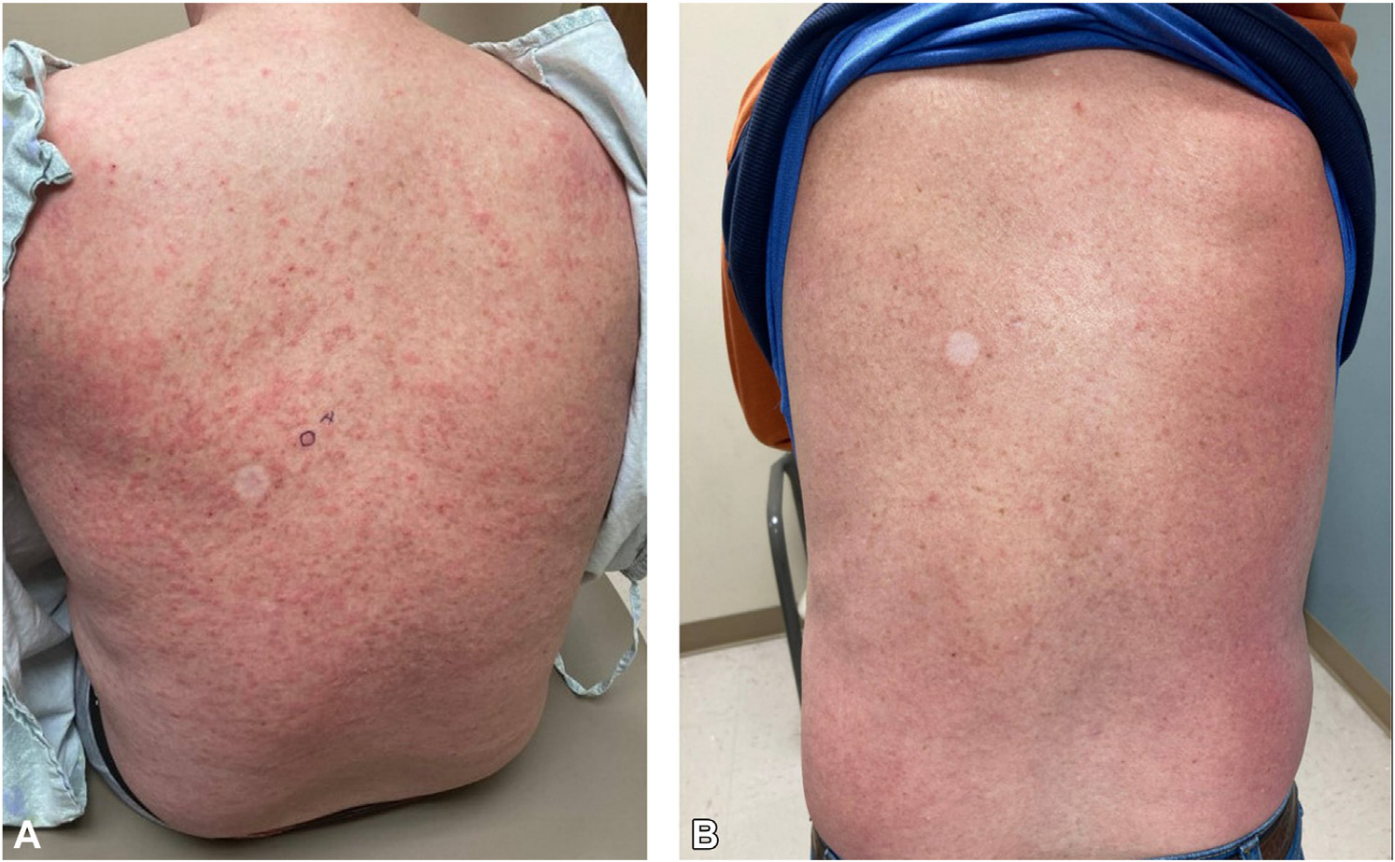
**A,** The initial reason this patient sought dermatology care was a pruritic truncal rash which was refractory to hydroxyzine and triamcinolone. Biopsy showed spongiotic dermatitis with eosinophils. **B,** After a year of intermittent treatment with triamcinolone, the truncal rash has improved, and pruritus continues to wax and wane.

**Fig 2:**
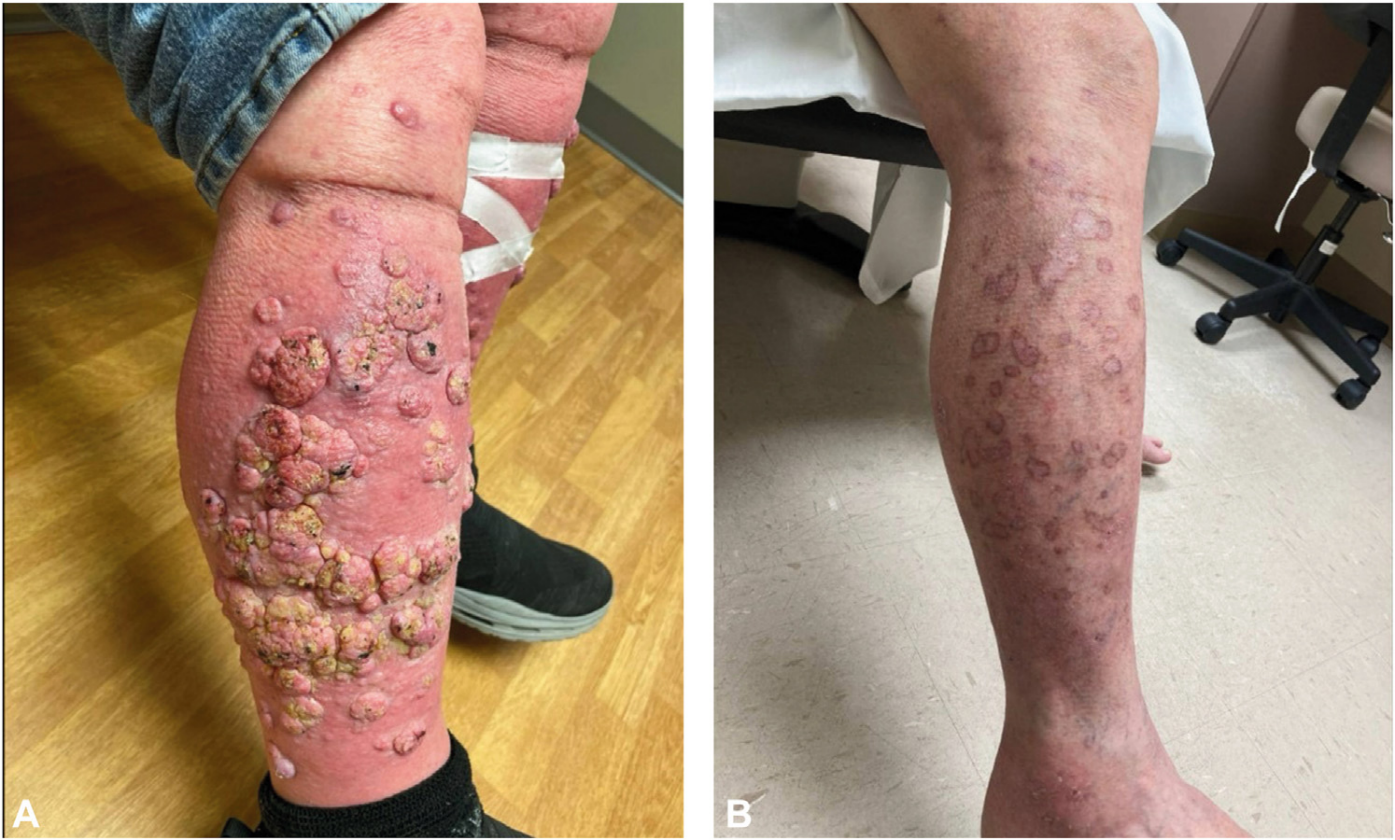
**A,** Exophytic papules and plaques on bilateral lower extremities. **B,** Resolving papules and plaques on bilateral lower extremities after treatment with intralesional fluorouracil injections, oral acitretin, and topical clobetasol.

A 64-year-old male was referred to the dermatology clinic with a chief complaint of a pruritic truncal rash in the setting of immunotherapy, comprised of minimally scaly red thin papules (Fig 1, *A*). Further exam of lower extremities revealed numerous exophytic plaques (Fig 2, *A*). Multiple prior biopsies of these lesions showed well-differentiated SCC which had been deemed metastatic and unresectable, thus leading to immunotherapy treatment.

This patient’s past medical history includes hypertension, coronary artery disease, and a 4 mm well-differentiated SCC successfully treated with Mohs 6 years prior. About 10 months previously, he had been diagnosed with SCC on his right lower leg which was surgically excised; he subsequently developed numerous similar lesions, with 6 biopsies on the lower extremities read as well-differentiated SCC. Initial positron emission tomography showed multifocal skin lesions and nonspecific pelvic nodes suspicious for metastatic disease; he initiated treatment with cemiplimab. A positron emission tomography scan 3 months later showed progression of the skin lesions and bilateral inguinal and external iliac hypermetabolic lymphadenopathy. He was subsequently started on a clinical trial of an antagonist of leukocyte immunoglobulin-like receptor B2 in combination with a programmed cell death protein 1 inhibitor and underwent 4 cycles of treatment. During this time, he also received palliative radiation to affected extremities.

He then presented to dermatology for diffuse pruritus and rash predominantly on his trunk, which had started around the same time that the eruptive nodules began. Further investigation included a biopsy showing spongiotic dermatitis with eosinophils, negative direct immunofluorescence, and a negative immunobullous disease antibody panel. The differential diagnosis included: id reaction in setting of diffuse eruptive squamous atypia (D-ESA), medication hypersensitivity, and contact dermatitis.

However, the diagnosis of unresectable cutaneous SCC was questioned due to the multifocal distribution on multiple extremities, prior histopathology showing well differentiation in all specimens, and lack of immunosuppression, or other risk factors for high-risk SCC. After an inguinal lymph node biopsy was requested and showed a benign reactive lymph node, it was determined that this presentation was more consistent with D-ESA rather than metastatic SCC. Clinical trial participation was discontinued, and we initiated treatment with acitretin (10 mg daily, later 20 mg daily), intralesional fluorouracil, and clobetasol.

The patient has received 11 rounds of intralesional fluorouracil to multiple lesions, and the plaques and swelling of his extremities have improved dramatically (Fig 2, *B*). While the truncal rash has improved, itching of his back continues to wax and wane despite treatment with triamcinolone (Fig 1, *B*). The patient declined repeat imaging of the pelvic lymph nodes but has remained without clinical adenopathy at the time of each check-up. We remain vigilant monitoring for signs of true SCC.

## Discussion

ESA, also known as eruptive keratoacanthoma, is a condition comprised of hyperkeratotic squamous proliferations with well-differentiated keratinocytes and low-grade histologic features. These idiopathic, sometimes koebnerizing, proliferations are often misdiagnosed as SCC or keratoacanthoma-type SCC.^2^, ^3^, ^4^ Here we report a unique case with an impressive presentation of D-ESA, initially misdiagnosed and treated with immunotherapy, which responded dramatically once recognized and treated as ESA.

These growths commonly occur at sites of previous surgical excision, trauma, tattoos, or radiation. However, synchronous eruption can also occur idiopathically.^2^^,^^4^^,^^5^ This condition more commonly occurs in female patients, with an average age of 69 to 73.5 years. Patients with ESA have had previous keratinocyte carcinomas and may or may not have a history of immunosuppression; these new growths are frequently located on the bilateral lower and upper extremities.^2^, ^3^, ^4^ In cases of ESA following trauma, the time to onset can vary from 6 to 16 weeks.^2^ In 2018 Que et al published an article with the first proposed clinical and histologic criteria and definitions as shown in Table I.^3^

**Table I:** ESA morphologies and descriptions as defined by Que et al^3^

ESA morphology types	Classification
Focal koebnerized ESA (FK-ESA)	At least 1 papule or plaque develops within 3 months of localized trauma, including after a clear-margin surgical excision
Diffuse ESA (D-ESA)	Multiple hyperkeratotic papules that develop concurrently

*ESA*, Eruptive squamous atypia.

Treatment for ESA is not well-defined, and various therapies reported in the literature are organized in Table II.^2^, ^3^, ^4^, ^5^, ^6^, ^7^

**Table II:** Treatment options for ESA

Treatment options for ESA
Radiation
Chemotherapy (topical, intralesional [IL]) *Fluorouracil (FU), methotrexate*
Retinoids [topical, oral] *Tazarotene, acitretin*
Immunotherapy [topical] *Imiquimod*
Curettage, electrodessication and curettage
Cryotherapy
Surgical excision
Mohs micrographic surgery

*ESA*, Eruptive squamous atypia.

Multiple case reports highlight that combination therapy often has better success than monotherapy.^3^^,^^4^ The most frequently reported therapies include a combination of oral retinoids and topical or intralesional chemotherapy. Que et al found that 67% of patients had resolution using intralesional-5 fluorouracil, and 33% of patients required additional therapy.^2^^,^^3^

Interestingly, certain literature highlights ESA as a side effect of programmed cell death protein 1 inhibitor therapy.^7^ Our patient did receive programmed cell death protein 1 inhibitor therapy, but the disease was already present prior to initiation of this therapy.

Our patient has responded well to the acitretin and intralesional 5-fluorouracil therapy. This unique case highlights that while there is a paucity of awareness of and research about ESA, nonsurgical combination therapy can be effective in treating D-ESA. As providers, it is important to recognize this rare diagnosis so patients are correctly classified, and they receive appropriate treatment.

## Conflicts of interest

None disclosed.
